# Evolutionary robotics as a modelling tool in evolutionary biology

**DOI:** 10.3389/frobt.2024.1278983

**Published:** 2024-11-05

**Authors:** Alan F. T. Winfield

**Affiliations:** Bristol Robotics Laboratory, UWE Bristol, Bristol, United Kingdom

**Keywords:** evolutionary robotics, evolutionary biology, synthetic method, robots as scientific instruments, model description

## Abstract

The use of evolutionary robotic systems to model aspects of evolutionary biology is well-established. Yet, few studies have asked the question, “What kind of model is an evolutionary robotic system?” This paper seeks to address that question in several ways. First, it is addressed by applying a structured model description developed for physical robot models of animal sensorimotor systems, then by outlining the strengths and limitations of evolutionary robotics for modelling evolutionary biology, and, finally, by considering the deepest questions in evolution and which of them might feasibly be modelled by evolutionary robotics. The paper concludes that although evolutionary robotics faces serious limitations in exploring deeper questions in evolutionary biology, its bottom-up approach to modelling populations of evolving phenotypes and their embodied interactions holds significant value for both testing and generating hypotheses.

## 1 Introduction

Evolutionary algorithms (EAs) are typically used to discover novel solutions to difficult design problems. Perhaps the most famous real-world example is an evolved satellite antenna design (Hornby et al., 2006). EA and its application in robotics, evolutionary robotics (ER), are thus seen as a technique for search or optimisation. However, evolutionary robotics may also be used to explore questions in evolutionary biology.

The idea of robots as scientific instruments is not new: Grey Walter’s electromechanical robot tortoises, *machina speculatrix*, were designed and built to test ideas in neuroscience. There is no doubt that Walter’s robots were the first biologically inspired robots (Holland, 2003b). The use of robotics as a comparative method in ecology and biology is well-established (Krause et al., 2011; Lauder, 2022), and as the literature surveys of Trianni (2014) and Doncieux et al. (2015) show, ER systems have also been used to model a wide range of interesting questions in evolutionary biology, including, notably, the co-evolution of predator–prey behaviour (Floreano and Nolfi, 1997), brain–body co-evolution (Lipson and Pollack, 2000), the evolution of altruism (Waibel et al., 2011), and the evolution of task specialisation of social insects (Ferrante et al., 2015). However, few works have sought to ask what kind of model an ER system is.

This paper proceeds as follows: Section 2 introduces the idea that robots can be scientific instruments. Section 3 outlines notable examples of experimental evolutionary robotics that have shed new light on the evolution of fish backbones, altruism, and modularity, exploring, with model descriptions, how ER systems model aspects of evolutionary biology before developing a critique of these models. Section 4 concludes with a discussion of deeper questions in evolutionary biology and whether they can or cannot be reasonably modelled with ER, as well as a set of recommendations for roboticists interested in studying evolutionary biology.

## 2 Robots as scientific instruments

The idea of robots that are not designed for their real-world utility but instead as scientific instruments is not new. If not the first, then certainly, the best-known example is W. Grey Walter’s *machina speculatrix*. Between 1948 and 1949, Walter, a neurophysiologist, designed and built two autonomous robots, which he named Elmer and Elsie. Their design was motivated by his theory of brain function, in particular that “cerebral functions may derive not so much from the number of [neurons], as from the richness of their interconnections” (Walter, 1953). Walter designed the robots’ control system with, as he put it, “a simple two-cell nervous system.” The ‘cells’ were vacuum tubes, and by variously connecting the cells with the robot’s sensors and motors, the robots demonstrated four distinct behaviours: exploration, obstacle avoidance, and both positive and negative phototaxis (Holland, 2003a).

**FIGURE 1:**
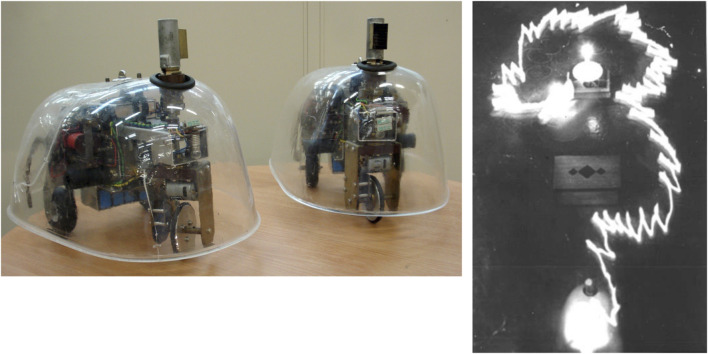
Left: Amy and Ninja, replicas of Walter’s machina speculatrix. Right: a time-lapse photograph of one of Walter’s 1949–50 experiments with Elsie showing obstacle avoidance and both positive and negative phototaxis (image: archive of the Burden Neurological Institute).

Figure 1 (left) shows two replicas of Walter’s robots built in the Bristol Robotics Laboratory by Ian Horsfield. In Figure 1 (right), we see one of Walter’s famous experiments, in which Elsie first moves toward a lamp, then ignores the lamp while avoiding an obstacle, and then shows positive phototaxis to move toward the lamp. When the robot gets close to the lamp, a combination of positive and negative phototaxis causes it to move around the lamp. Arguably, the two cells are equivalent to what we would now call a single-layer recurrent artificial neural network. The analogy is apt, given that Walter’s design fully exploited the non-linear properties of the vacuum tubes. He reportedly “…stressed the importance of using purely analogue electronics to simulate brain processes at a time when his contemporaries such as Turing and von Neumann were all turning toward a view of mental processes in terms of digital computation”^1^.

The fact that Walter’s *machina speculatrix* are robots built with purely scientific aims is not an accident. Walter was well aware of the *synthetic method*, a term employed by the contemporary psychologist Kenneth Craik “to describe the process of testing behavioural theories through machine models” (Bisig et al., 2008). Walter himself wrote: “In general, it is legitimate to study a model of a mysterious process if three conditions are fulfilled: 1. several features of the mystery must be known. 2. The model must contain the absolute minimum of working parts to reproduce the known features. 3. The model must reproduce other features, either as predictions or as unexpected combinations” (Walter, 1953, 280).

In recent decades, the synthetic method, in which robots are used as working models, has become well-established in the study of both animal behaviour and physiology: an approach that has become known as either artificial ethology (Holland and McFarland, 2001) or simply biorobotics (notably, the term biorobotics also includes biologically inspired or biomimetic robots). Examples include the landmark work on cricket phonotaxis by Webb (1995) and the work on collective sorting and segregation by Melhuish et al. (1998). However, the use of evolutionary robotics for modelling aspects of evolutionary biology is much less well-established.

### 2.1 Related work

There are few works that critically examine the potential of ER as a tool for addressing questions in evolutionary biology, although the rationale for doing so was neatly articulated by Maynard Smith in 1992: “so far, we have been able to study only one evolving system, and we cannot wait for interstellar flight to provide us with a second. If we want to discover generalizations about evolving systems, we have to look at artificial ones” (Maynard Smith, 1992).

In ‘Evolutionary robotics: model or design?’ Trianni (2014) offered, perhaps, the first thorough review and critique of evolutionary robotics as a modelling tool for biology. Trianni makes the case that ER uniquely provides us with a bottom–up model of an evolving population of model organisms that allows us to “identify the causal relationship between selective pressures and adaptive traits, thanks to the possibility of having complete control over the evolutionary process.” In ‘Evolutionary robotics: what, why, and where to,’ Doncieux et al. (2015) asked the question “Evolutionary robotics, for whom?” and addressed biologists, asserting that “evolutionary robotics provides tools for modelling and simulating evolution with unique properties: considering embodied agents that are located in a realistic environment makes it possible to study hypotheses on the mechanistic constraints at play during evolution” that are “particularly relevant for modelling behaviours where complex interactions within the group and with the environment are at work.”

This paper aims to build upon and complement these works by addressing the question, “What kind of model is an ER system?” This question is explored by outlining three notable ER models of evolutionary biology alongside model descriptions using the approach proposed by Webb (2001) (introduced below), and by doing so, this paper addresses Eiben (2021)’s call for “instruments to formally describe and analyse evolutionary robot systems.” The subsidiary contributions are as follows: (i) a proposed extension to Webb’s model for ER, (ii) an analysis of the strengths and limitations of ER for modelling evolutionary biology, (iii) a review of the major transitions in evolution and which of these might be addressed by ER, and (iv) recommendations for roboticists proposing to model biology with ER.

## 3 What kind of model is an evolutionary robotic system?

In seeking to address this question, a good place to start is Barbara Webb’s paper titled ‘Can robots make good models of biological behaviour?’ (Webb, 2001), which focuses on building physical robot models of biological sensorimotor systems in order to investigate problems in biology. The paper sets out a framework with seven dimensions for comparing robot models of biological behaviour as a structured approach for evaluating and comparing different modelling approaches in the context of biological systems. Webb concluded that “a dimensional description should not be primarily considered as a means of ranking models as ‘better’ or ‘worse’ but rather as an elucidation of potential strategies,” with a strategy of “increasing relevance and commitment to really testing biological hypotheses; …aspiring to accuracy but concerned with building complete systems; looking for a closer behavioural match; and using real physical interaction as part of the medium.” Webb’s seven dimensions, the questions they each address, and a commentary/interpretation are given in Table 1.

**TABLE 1:** Webb’s seven dimensions for describing models. The dimensions and questions are from Webb (2001). For each dimension, an interpretation has been added.

Dimension	Question and interpretation
Relevance	Is the biological target system clearly identified?
	*Relevance* focuses on how well the model represents a real biological system and whether it has the potential to test or generate hypotheses relevant to biology
Level	What are the base units of the model?
	This dimension specifies the organizational *level* at which the model operates. It might, for instance, be a limb or sense organ, neuronal, the whole organism, or a complete ecosystem
Generality	How many systems does the model target?
	*Generality* refers to the extent to which a model applies to a wide range of real-world systems or biological phenomena
Abstraction	What is the complexity of the model or amount of detail included, relative to the target?
	*Abstraction* assesses the number and complexity of mechanisms included in the model; a more detailed model is less abstract. Webb notes that “a simple target might be represented by a simple, but not abstract, model, and a complex model still be an abstraction of a very complex target”
Structural accuracy	How well does the model represent actual mechanisms underlying the behaviour?
	*Structural accuracy* focuses on the fidelity of the model in reflecting the real mechanisms and processes that drive the observed behaviour in the target biological system. We would assess structural accuracy by evaluating how closely the mechanisms included in the model mirror the mechanisms operating in the target system
Performance match	To what extent does the model behaviour match the target behaviour?
	This dimension describes how well the model replicates the observed behaviours, responses, and outcomes of the biological system under study. A high level of *performance match* would indicate that the model accurately captures the essential characteristics and dynamics of the target system’s behaviour
Medium	What is the physical basis by which the model is implemented?
	*Medium* describes the physical basis by which the model is implemented. This could be computer simulation or physical robot(s) in some test environment or a hybrid of the two

Let us now review three notable examples of artificial evolutionary systems that have addressed questions in evolutionary biology. These three case studies have been chosen to represent very different levels of generality, abstraction, and structural accuracy. They also cover a very broad range of targets: physical traits of aquatic vertebrae, collective behaviour within a model ecosystem, and the evolution of modularity. For each of these studies, a description using Webb’s dimensions is made in order to compare and contrast the three models.

### 3.1 The evolution of fish biomechanics

John Long is well known for his use of evolutionary robotics to investigate the biomechanics of fish (Long, 2012). We consider his work using ER to test the hypothesis that vertebrae in ancient fish “evolved as a locomotor adaptation, stiffening the body axis and enhancing swimming performance” (Long et al., 2006). Long and his team extended biomimetic evolutionary analysis (BEA), which builds physical simulations of extinct systems, to include the use of autonomous robots as models of early vertebrates competing in a foraging task. They designed a biomimetic tadpole robot, called *Tadro*, with a biomimetic tail. *Tadro* has a single eyespot (photoresistor), a flapping tail, and a controller that converts the light intensity at the eyespot into a turning angle at the tail.

In this work, three physical Tadro robots compete to reach and encircle a light source (the food target) in a water tank. The robots’ fitness, called navigational prowess (NP), was rewarded by four measures: short time to reach the target, small orbital radius, fast swimming speed, and low robot wobble. The genome coded for two parameters, the robot’s flapping tail bending modulus and length, which together determine stiffness. Following selection, crossover, and mutation, the next-generation robot’s tails (biomimetic notochords) were fabricated by hand with cylindrical hydro-gels formed from gelatine (analogous to individual vertebrae), which were then connected by a flexible glue (analogous to inter-vertebral discs). Long et al. (2006) concluded that “we see evidence to support the classical hypothesis that vertebrae stiffen the body and that increased tail stiffness increases thrust production and steady swimming speed,” although interestingly adding the rider that “this formulation, however, avoids a central evolutionary question: under what ecological and selective conditions might vertebrae evolve?”.

Table 2 describes Long’s model using Webb’s dimensions. Here, we see that the biological relevance is high with a clearly identified target: the evolution of vertebrae in ancient fish. The level, or unit, of the model is very specific: a backbone with variable stiffness. The model is not abstract and has relatively low complexity, yet it has sufficient structural accuracy to model the effect of vertebrae stiffness on swimming, thus both supporting the classical hypothesis and providing a good performance match.

**TABLE 2:** Description of Long et al. (2006) using Webb’s model dimensions of Table 1.

Dimension	Description
Biological relevance	The target is clearly identified: the evolution of vertebrae in ancient fish
Level	Backbone with variable stiffness and tail
Generality	The range of animals modelled is large: aquatic vertebrata
Abstraction	The model is not abstract, and its complexity is relatively low
Structural accuracy	High: sufficient accuracy to model effect of vertebrae stiffness on swimming
Performance match	Good: the classical hypothesis is supported, with additional insights
Medium	Physical robots swimming toward a beacon in a water tank

### 3.2 The evolution of cooperation and altruism

Next, we consider the work of Waibel, Floreano, and Keller, which made use of the miniature ALICE robots to evolve cooperative and altruistic behaviours (Waibel et al., 2009; Waibel et al., 2011). In these experiments, a group of robots forage by finding and collecting physical tokens and moving them to a nest area. The experimental arena contained two kinds of tokens: small tokens that can be pushed by a single robot and larger tokens too heavy to be pushed by a single robot. The fitness of each robot was based on its success in foraging for tokens. In one experiment, the arena contained only large tokens, and the robots successfully evolved the ability to cooperatively push these tokens to the nest.

However, when the arena contained both large and small tokens, the group ‘kin structure,’ i.e., the genetic relatedness of the individuals, influenced the evolved behaviours. Groups of genetically unrelated robots evolved toward pushing the small tokens because this was the best way to maximize their own fitness. In contrast, related robots evolved altruistic behaviours—cooperating to push the large tokens at the expense of their own individual fitness. The result quantitatively confirms Hamilton’s rule that altruistic behaviours will evolve when the relatedness of individuals multiplied by the fitness benefit to the receiver of the altruistic behaviour is greater than the fitness cost of performing the behaviour (Bourke, 2014). See Table 3 for a description of Waibel et al. (2009)’s model using Webb’s dimensions, in Table 1.

**TABLE 3:** Description of Waibel et al. (2009) against Webb’s model of Table 1.

Dimension	Description
Biological relevance	Medium: the collective behaviours of cooperative groups of social animals
Level	Simple model ecosystem
Generality	Range of biological systems modelled is wide: cooperating social animals
Abstraction	This is an abstract model of a medium-complexity target system
Structural accuracy	Sufficient accuracy to model the emergence of both cooperation and altruism
Performance match	High, given the model’s quantitative confirmation of Hamilton’s rule
Medium	Ten physical robots foraging two sizes of food token in an arena

In contrast with Table 2, here, Waibel et al. (2009)’s model is abstract, with a lower level of biological relevance as no specific animals are modelled. Instead, in Table 3, the general class of cooperative groups, or social animals, is modelled. The level (or unit) of the model is a simple model ecosystem, in which each individual is modelled with a mobile robot. In common with Long et al. (2006)’s model in Table 2, the generality, structural accuracy, and performance match are all high, demonstrating that lower biological relevance and higher abstraction do not limit the value of the model.

### 3.3 The evolution of modularity

Our third case study is concerned not with the evolution of particular traits but instead the evolution of modularity. It is well known that biological evolution is highly modular. Complex organisms are assemblages of pre-evolved components; cells are ubiquitous building blocks for evolution; and organs such as eyes, hearts, and livers or sub-systems such as vascular or digestive systems are themselves modules that are re-used with often surprisingly little change across species within a given taxonomic class (e.g., Mammalia) (von Dassow and Munro, 1999).

**FIGURE 2:**
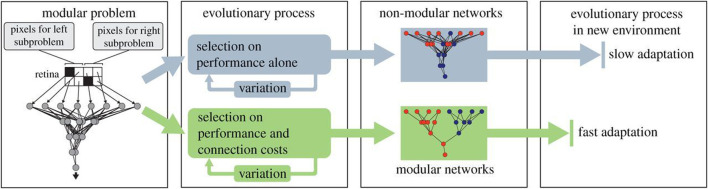
Two evolutionary processes: upper, based on performance alone; lower, based on both performance and connectivity costs. Diagram from Clune et al. (2013), with permission.

With an elegant application of the evolutionary algorithm, Clune et al. challenged the sole focus on selective forces as the origin of biological modularity (Clune et al., 2013). Unlike the two previous works outlined in this section, Clune et al. are not concerned with physical robots. Instead, they evolve the controller, an artificial neural network, of a conceptual creature in simulation. The ANN evolves to recognize patterns (objects) in a simple eight-pixel retina. Importantly, the problem is modularly decomposable because whether an object exists on the left or right sides can be separately determined before combining that information to determine whether objects exist on both sides. Evolving the ANN with selection for performance alone resulted in non-modular networks that are slow to adapt to new environments. However, the simple addition of a second selection pressure to reduce connectivity in the ANN led to the evolution of modular networks that show faster adaptation in new environments than the non-modular networks, as illustrated in Figure 2.

The biological relevance of Clune et al. (2013)’s model in Table 4 using Webb’s classification is arguably low as no general class of animals is modelled here. Yet, the model remains highly relevant as even the simplest animals exhibit modularity, and the model has the highest generality of all three: the evolution of evolvability, which is “the ability of a biological system to produce phenotypic variation that is both heritable and adaptive” (Payne and Wagner, 2019). This model is highly abstract with a low structural accuracy, yet, in common with all three models, it shows a good performance match.

**TABLE 4:** Description of Clune et al. (2013) using Webb’s model dimensions.

Dimension	Description
Biological relevance	Evolution of modularity.
Level	Neural controller (ANN)
Generality	Very high: the evolution of evolvability
Abstraction	Highly abstract: a minimal model of the evolution of modularity
Structural accuracy	Low but sufficient; no real animals are modelled
Performance match	Good: model demonstrates a selection mechanism that evolves modular ANNs
Medium	Computer simulation

### 3.4 Extending Webb’s model description for evolutionary robotics

**FIGURE 3:**
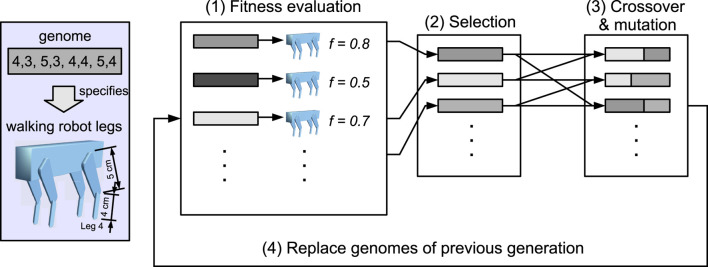
Four-stage process of evolutionary robotics, which depicts a quadrupedal robot where the length of the upper and lower leg segments of each leg is evolved; from Winfield (2012).

It is clear that the example model descriptions in Tables 2–4 are incomplete. There are several important dimensions present in ER models that are not covered by Webb’s model description. This is hardly surprising, given that Webb’s model description was developed primarily with single-robot models in mind. ER also has robot models, but as Figure 3 shows, it additionally evolves a population of phenotypes, which involves genotype to phenotype mapping, selection, and fitness evaluation.

Table 5 proposes several additional dimensions, which, together with Webb’s model, would allow us to provide a more complete description of ER models. The first three dimensions in Table 5 simply provide the number of *gene pools*, *population sizes*, and the *number of generations* in a run of the ER model. In the study of Long et al. (2006) of Section 3.1, there is one gene pool, with a population of three phenotypes, and the ER model was run for 10 generations. In contrast, the study of Waibel et al. (2009) of Section 3.2 has a population of 1,000 organised into teams of 10 robots each, and the model was run for 300 generations. These details should be included in the model description.

**TABLE 5:** Proposed set of additional ER model dimensions.

Dimension	Interpretation
Gene pools	How many separate gene pools (i.e., ‘species’) are modelled?
Population size	What is the size of each gene pool?
Generations	How many generations are evolved in a run of the ER model?
Mapping	Is the genotype to phenotype mapping direct or indirect? If indirect, give detail
Brain/body	Does the ER model evolve the brain, body, or both? Which parameters?
Fitness function(s)	What do fitness function(s) reward?
Selection strategy	What selection strategy is used to select the fittest individuals?
Evo or evo-devo	Does the ER model incorporate developmental learning?

The *mapping* dimension in Table 5 concerns the mapping from genotype to phenotype, which may be direct or indirect. If indirect, then the description should provide the details. Doncieux et al. (2015) provided a good description of genotype to phenotype mapping. *Brain/body* very simply describes whether the ER model evolves the controller alone, the body morphology alone, or both. Our sixth proposed new dimension specifies the *fitness function* (or functions if there is more than one co-evolving species). For a survey of fitness functions in ER, see Nelson et al. (2009). *Selection strategy* specifies the method used to select the fittest individuals. For a survey of selection strategies, see Fernández Pérez et al. (2014).

The final dimension is concerned with evolutionary developmental biology, known by the shorthand ‘evo-devo’ (Nuño de la Rosa and Müller, 2020). In recent years, evo-devo has become important in ER, and some argue that “if it evolves, it needs to learn” (Eiben and Hart, 2020). There have been a number of very elegant experimental studies in artificial evo-devo. Kriegman et al. (2018), for instance, evolved, in simulation, a population of soft-bodied robots each consisting of a 4 x 4 x 3 grid of voxels. The soft robots evolved the ability to move over a flat terrain as quadrupeds, but, interestingly, sometimes, they learnt a new rolling morphology during their lifetime. Although not modelling biology, Kriegman et al. (2018) showed that “development, under the right conditions, can increase evolvability.” Should such evo-devo systems be developed to model evo-devo in biology, then the final dimension in Table 5 *evo-devo* will be needed in the model description.

As an example, Table 6 shows an extended model description for the work of Long et al. (2006), as outlined in Section 3.1 above, demonstrating the value of extending Webb’s model description for ER in providing a much more complete picture.

**TABLE 6:** Description of Long et al. (2006) using the extended model description proposed here.

Dimension	Description
Biological relevance	The target is clearly identified: the evolution of vertebrae in ancient fish
Level	Backbone with variable stiffness and tail
Generality	The range of animals modelled is large: aquatic vertebrata
Abstraction	The model is not abstract, and its complexity is relatively low
Structural accuracy	High: sufficient accuracy to model effect of vertebrae stiffness on swimming
Performance match	Good: the classical hypothesis is supported, with additional insights
Medium	Physical robots swimming toward a beacon in a water tank
Gene pools	1
Population size	3
Generations	10
Mapping	Direct
Brain/body	Body: tail, two parameters: bending modulus and length
Fitness function	Min time to target, min orbital radius, max swim speed, and min robot wobble
Selection strategy	Selection based on fitness and random combination to produce a new generation
Evo or evo-devo	Evo. There is no developmental learning

### 3.5 Observations, strengths, and limitations

Consider the schematic description of a complete ER system in Figure 3. All three of the case studies above follow the broad approach of Figure 3. The work of Long et al. (2006) in Section 3.1 evolves the robot morphology, as in Figure 3, whereas the works of Waibel et al. (2011) and Clune et al. (2013) evolve only the robots’ controller ANNs, so the genomes specify connection weights rather than physical properties.

Figure 3 prompts two important observations. The first is that the model depicted here is a high-level macro model, with several sub-models. The three Darwinian operators: *selection*, *variation*, and *heredity* are modelled here. Stages (1) and (2) model selection, stage (3) models variation, and stage (4) models heredity. All evolution requires a population of conspecifics, and this population is modelled in all four stages. Another sub-model present here is the model genome, and the phenotype instantiated from a single genome is—if we are modelling biology—a model of an animal. Notably, in this illustration, the genotype (complete set of genetic material) consists of just one genome (or more correctly, an *allele*) as the phenotype is fixed, with the exception of only the eight upper and lower leg segment lengths.

A second and key observation is that ER does not model natural selection. As Eiben and Smith (2015) put it, “evolutionary algorithms are not faithful models of natural evolution.” Rather, ER models *artificial selection*. This is neither surprising, given that the whole idea of ER is to discover novel robot designs, nor is it a bad thing. The first four chapters of *On the Origin of Species* lay out Darwin’s case that selection in nature, driven by the struggle for existence, is analogous to the selection of variations under domestication (Darwin, 1859). Human farmers and herders have been successfully cultivating improved varieties of grains, vegetables, and animals for at least 10,000 years; a process that was very well-understood in Darwin’s time. Figure 3 is thus more accurately described as a process of *robot husbandry*, and therefore, it is a putative model of animal husbandry. The case studies above clearly demonstrate that an artificial selection/animal husbandry model can be used to test hypotheses in evolutionary biology.

Open-ended evolution has been the subject of investigation of several studies in ER. Bianco and Nolfi (2004), for instance, proposed a framework for an open-ended evolutionary process in hardware, noting that, “In the case of natural evolution, there are no selection criteria that determine whether individuals can or cannot reproduce aside from the ability to reproduce itself.” Bredeche and Montanier (2012) described an experimental work in simulation and with real robots that demonstrates environmental-driven, distributed, and open-ended evolution. Although of great interest, this work is not offered as an ER model of natural selection in biology, which remains a very significant challenge.

We now consider the strengths and limitations of ER, as specified in Figure 3, as a model of evolutionary biology.

#### 3.5.1 Strengths

1. When modelling is the only option: as Trianni (2014) and Doncieux et al. (2015) argue, ER models allow us to explore questions in evolutionary biology that would not be possible with living animals or ecosystems. Trianni (2014) stated that “ER is especially fruitful when it is impossible or unpractical to run experiments directly with the biological system, either as laboratory or field work.”
2. Multiple ‘species’: ER allows us to co-evolve the behaviours of different ‘species’ within a single ER model by providing two (or potentially more) gene pools. It is important to stress that these are separate species by design, when, in the model, we do not allow interbreeding across the gene pools. One example is given in Section 3.2 above, with the evolution of cooperation and altruism. Another great example is the work of Floreano and Nolfi (1997), which demonstrates the co-evolution of predator–prey behaviour. Note that two or more species in a single ER model also requires two or more corresponding fitness functions. Floreano and Nolfi (1997) maintained a population of 100 predators and 100 prey, which were subject to selection for successful predation and predator evasion, respectively.
3. The richness of matter: when ER systems make use of real physical robots for fitness testing, as in the first two case studies above, the ER model benefits in two important ways. (i) Real robots must contend with the physics of the real world. This is especially relevant in Long et al. (2006)’s work, as outlined in Section 3.1 above. The real robots have to float and swim in water, the same medium as the fish the robots are modelling. Any effort to simulate the same swimming robots would invariably suffer a loss of fidelity, given the difficulty of simulating the complex dynamics of the interactions between the tail and water. With real robots in a real medium, all of that complexity comes for free. (ii) When several real robots interact during fitness testing, as in the work of Waibel et al. (2009) outlined in Section 3.2 above, the model benefits because each robot is slightly different. The small heterogeneities of robots that have slightly different motors, wheels that might not be precisely aligned, and sensors that are not identical, etc., add a degree of stochasticity that, although not identically, models the heterogeneities of animal conspecifics. This adds valuable richness to these ER models.
4. Controllable conditions: a clear benefit of an ER model is that the initial conditions of the model are fully specified, which include the initial phenotype(s), selection, crossover and mutation processes, fitness function(s), and the fitness testing environment. This provides a high degree of repeatability. Inevitably, if fitness testing makes use of real robots, then, as outlined above, the stochasticity of the environment and the physical robots will likely alter the course of evolution across successive runs from identical starting conditions. Arguably, this is a feature of an ER model rather than a bug, given that biological evolution is both contingent and “surprisingly repeatable among closely related lineages” (Blount et al., 2018).

#### 3.5.2 Limitations

1. Sparse environment: Most animals evolve in a complex and dynamic environment that goes through cyclic changes: day–night and seasonal changes plus occasional traumatic variations. Yet, in ER fitness evaluation, step (1) in Figure 3 typically takes place in a static test arena, with simple features designed to test the robots’ fitness. Only when co-evolving cooperative or antagonistic behaviours is there a semi-dynamic environment, in that one ‘species’ of moving robot(s) provides an environment for the other(s), but this falls far below the level of richness of real-world ecological niches. We know that the environment strongly influences natural selection, but we know little about how it does so (Payne and Wagner, 2019). There have, however, been ER studies that focus on the way the environment influences robot morphology. Miras et al. (2020), for instance, evolved multi-segmented modular robots for locomotion on both flat and tilted surfaces, and Corucci et al. (2018) evolved soft robots in simulation for both terrestrial and aquatic environments and the transition between them while also varying the material properties of the soft robots. Although not offered as a model of evolutionary biology, the latter work does offer potentially valuable insights. Corucci et al. (2018) reported an asymmetry in the effects of moving between land and water: “while moving from land to water resulted to be detrimental for the evolution of swimming, the opposite transition (land to water) pointed out some benefits for the evolution of walking.”
2. Small evolutionary distance: If, as suggested above, ER is a process of robot husbandry, then it is axiomatic that there needs to be an initial population of robots from which better (or different) robots can be bred. We, therefore, need to hand design the phenotype(s) of the first generation with properties, including sensing, actuation, and a control system, that appropriately model the kinds of animals that are the subject of our inquiry. This means that there is typically a very small evolutionary distance, as a result of the small population size and number of generations (relative to biological evolution), between the initial designed population and the final population at the point we choose to halt the ER model. One important corollary is that evolutionary branching, the “spontaneous transition from a unimodal trait distribution into a bimodal one,” is almost impossible when the “effective population size or mutational effect is sufficiently small” (Wakano and Iwasa, 2012). As the example from Long et al. (2006) outlined in Section 3.1 above shows, ER cannot model the evolution of a backbone but only the evolution of a better backbone.
3. Weak genotype to phenotype mapping: In evolutionary biology, it is now well known that the ‘genes as blueprint’ metaphor is not only inadequate but also misleading (Pigliucci, 2010). In an excellent survey of open issues in ER, Silva et al. (2016) reported that the majority of ER studies use direct encoding; they also make the important point that indirect encodings “enable representational efficiency … by incorporating concepts from evolutionary developmental biology.” In recent years, compositional pattern-producing networks (CPPNs) have become popular (Cheney et al., 2013). For an analysis of the strengths and weaknesses of CPPNs, see Silva et al. (2016). CPPNs, although more biologically plausible than direct mapping, do not constitute a model of genotype to phenotype mapping in evolutionary biology. For a recent account of genetic representations for co-evolving behaviour and morphology in ER, see De Carlo et al. (2024).
4. The reality gap: Given the time and resource cost of physically building robot phenotypes for fitness testing, a popular approach in ER has been to simulate multiple generations of evolution in software until robots judged fit enough have evolved, and then to build and test those. This strategy, known as ‘simulate and transfer to real,’ is efficient but suffers from the well-known reality gap (Jakobi et al., 1995). For a very good overview of the many subsequent approaches to crossing the reality gap, see Silva et al. (2016). When ER is modelled entirely in hardware, as in Long et al. (2006), there is no gap. However, a hardware-only ER model is not only very costly in time and resources but also reduces the evolutionary distance (limitation 2) still further. Thus, hybrid software ER models into which hardware phenotype instantiation and fitness testing are periodically intercalated are preferred (Eiben et al., 2021). Evolution is an extremely energy and resource-intensive process (Winfield, 2014), and thus, it should come as no surprise that ER models are also costly in time and resources.

## 4 Concluding discussion

Clearly, we can use ER to ask interesting but straightforward questions relative to the complexity of many questions in evolutionary biology, as shown in the case studies in Section 3 above. However, what questions in evolutionary biology are too difficult to address with an ER model?

Consider Maynard Smith and Szathmáry’s seminal work, *The Major Transitions in Evolution* (Maynard Smith and Szathmáry, 1995). Might ER be able to model any of the eight transitions set out in that work? The first three transitions: (1) from replicating molecules to populations of molecules in proto-cells, (2) from independent replicators to chromosomes, and (3) from RNA to DNA, are clearly out of scope of ER, in which we hand-design the replicator. Transition (4) from prokaryotes to eukaryotes is also out of scope, given that this transition is concerned with the evolution of the structure of single-celled organisms. Transition (5) is the evolution of sex, from asexual clones to sexual organisms (most single-celled eukaryotes reproduce sexually). Modelling this transition is problematic because sexual reproduction remains a mystery in evolutionary biology, described as both a paradox and the “queen of problems” (Bell, 1982; MacPherson et al., 2023). However, a recent work describing a real-robot ER system with mutable diploid genes, which models death, rebirth, and breeding in a stochastically varying landscape, hints at the potential of ER for modelling the emergence of sexual reproduction (Wang et al., 2022)^2^.

Transition (6), the evolution of multicellularity, could, in principle, be modelled by combining modular robotics (in which robot modules model cells) and ER. The Symbrion project, for instance, did combine collective, modular, and evolutionary robotics within a single framework (Schlachter et al., 2008). In Symbrion, individual modules, when acting as a swarm, represented stem cells. When triggered by one robot encountering an environmental cue (such as an obstacle too high to climb), that robot initiates the formation of a ‘multi-cellular’ organism, in which each “cell” differentiates in order to behave in a specific way, according to its physical location in the organism (Liu and Winfield, 2010). However, this process of autonomous morphogenesis was programmed, and the technical complexity meant that modelling the evolutionary emergence of multicellularity was impossible. The first two limitations outlined in section 3.5 above, the sparse environment and short evolutionary distance, will almost certainly thwart any efforts to model the evolution of multicellularity. Evolutionary developmental soft robots may, however, provide a pathway to modelling transition (6) (Corucci et al., 2017).

The same two limitations may also prevent ER from feasibly modelling transition (7), the evolution of eusociality (colonies with non-reproductive castes). However, one evolutionary swarm robotics study has notably demonstrated the emergence of one aspect of eusociality: task specialisation in social insects. Ferrante et al. (2015) reported a surprising result that division of labour “could be achieved merely by selecting on overall group performance and without pre-specifying how the global task of retrieving items would best be divided into smaller subtasks.” In addition to advancing the field of evolutionary swarm robotics, the work of the paper offers a possible explanation for the origin of division of labour in social insects.

Transition (8) is the evolution of culture from primate societies to human societies with language and culture. Since this transition concerns behavioural or memetic evolution, it does not require an ER model as the phenotype does not need to evolve. A relatively simple model in which a group of real robots demonstrate the evolution of new behavioural traditions has been developed (Winfield and Blackmore, 2022). In this model, the three Darwinian operators of selection, mutation, and heredity are present, but rather than genes, it is behaviours (memes) that are selected and mutated across successive generations of meme.

Consider also niche construction. Biological complexity apparently arises from an evolutionary arms-race, in which organisms both adapt to and exploit niches in their ecosystem and, in doing so, co-create that ecosystem. As Levins and Lewontin (1985) pointed out, the organism is both the subject and the object of evolution. Niche construction is the process by which organisms continuously modify their own and others’ niches; from a niche construction perspective, “evolution consists of mutual and simultaneous processes of natural selection and niche construction” (Laland et al., 2000). The sparse environment limitation almost certainly rules out ER as a model of niche construction.

The discussion above appears to rule out ER as a method for modelling the deepest questions in evolutionary biology. However, provided that we fully understand their limitations, ER models can, and have already, addressed interesting questions in evolutionary biology. Within the scope are questions on how particular traits evolve in particular kinds of animals. It also includes questions on how interaction between different ‘species’ might evolve. In addition to the predator–prey behaviour of Floreano and Nolfi (1997) and cooperative behaviours of Waibel et al. (2011), we might be able to model symbiosis, mutualism, or even parasitism. More abstract questions that might also be within scope include the dynamics of brain–body evolution or the trade-offs between morphological and computational intelligence (Zahedi and Ay, 2013). As Trianni (2014) writes, “In the ER context, considerable caution is needed, given that artificial evolution is a very simplified model of natural evolution:” he also suggests that it is “…wrong to *a priori* proscribe ER as a modelling tool, but it is necessary to evaluate case by case whether the proposed ER model can be of some value.”

To that end, several recommendations follow. Roboticists proposing to use ER to study aspects of evolutionary biology should ideally:

1. Have a reasonable understanding of evolutionary biology^3^.
2. Given that framing research questions in evolutionary biology is itself not straightforward, roboticists should work with evolutionary biologists, either as member(s) of the research team or as advisor(s). In particular, the biologist(s) would guide the framing of the research question and its context within evolutionary biology.
3. Following Trianni (2014), adopt a hypothesis-driven rather than exploratory approach, in which the whole experimental design is conceived for the purpose of testing or generating hypotheses^4^.
4. Take great care to fully understand the properties and limitations of the model they are building using, for instance, the case studies of Section 3 and Webb’s extended model description in Section 3.5 above.
5. Employ the principle of *ceteris paribus* by fixing all but one of the subsystems of an ER system and their parameters. The only parameters that should be allowed to vary are coded in the genome, the very parameters that are evolving.
6. Resist the temptation to use the same ER system to evolve real-world-useful robots while also seeking answers to questions in evolutionary robotics. ER systems cannot be used as scientific instruments and discoverers of novel robots for the real world at the same time.

In conclusion, it is clear that ER is a weak model of evolutionary biology. Its primary weakness is that essentially, ER models artificial selection not natural selection. This combined with sparse model environments, typically small population sizes and number of generations leading to a small evolutionary distance and weak genotype to phenotype mapping, mean that the level of abstraction of ER models is often high. These weaknesses impose serious limitations on the bigger questions in evolutionary biology that can be feasibly explored in a way that has value. Despite these manifest weaknesses, ER’s bottom–up approach of modelling populations of evolving phenotypes and their embodied interactions with each other and their environment does have considerable value for biologists for both testing and generating hypotheses.

Using evolutionary robotics to rigorously address research questions in evolutionary biology is without doubt the most challenging application of robots as scientific instruments to date. Rising to this challenge is certainly a worthy goal.

## Data Availability

The original contributions presented in the study are included in the article; further enquiries can be directed to the corresponding author.

## Footnotes

1
https://en.wikipedia.org/wiki/Elmer_and_Elsie_(robots)

2 The authors propose that “diploid gene robots can act as avatars of diploid mammalian cells to explore novel programs of administration of drugs.”

3 For recommended reading on evolution, see https://www.sciencefocus.com/books/best-evolution-books/

4
Trianni (2014) warns that “exploratory studies (in which experiments are conducted to understand the possibilities offered by robotics and ER as a modelling tool) are the most susceptible to find themselves in the limbo between modelling and design.”
